# Coagulative Disorders in Critically Ill COVID-19 Patients with Acute Distress Respiratory Syndrome: A Critical Review

**DOI:** 10.3390/jcm10010140

**Published:** 2021-01-03

**Authors:** Chiara Robba, Denise Battaglini, Lorenzo Ball, Alberto Valbusa, Italo Porto, Roberta Della Bona, Giovanni La Malfa, Nicolò Patroniti, Iole Brunetti, Maurizio Loconte, Matteo Bassetti, Daniele R. Giacobbe, Antonio Vena, Claudia Lucia M. Silva, Patricia R. M. Rocco, Paolo Pelosi

**Affiliations:** 1 Department of Anesthesia and Intensive Care, Ospedale Policlinico San Martino, IRCCS for Oncology and Neuroscience, 16132 Genoa, Italy; battaglini.denise@gmail.com (D.B.); lorenzo.loryball@gmail.com (L.B.); nicolo.patroniti@unimib.it (N.P.); brunettimed@gmail.com (I.B.); maurizio.loconte15@gmail.com (M.L.); ppelosi@hotmail.com (P.P.); 2 Department of Surgical Sciences and Integrated Diagnostics (DISC), University of Genoa, 16132 Genoa, Italy; 3 Dipartimento CardioToracoVascolare, Ospedale Policlinico San Martino IRCCS, 16132 Genoa, Italy; albertovalbusa@yahoo.it (A.V.); italo.porto@gmail.com (I.P.); roberta.dellabona@gmail.com (R.D.B.); g.lamalfa4@gmail.com (G.L.M.); 4 Dipartimento di Medicina Interna e Specialità Mediche (DIMI), University of Genoa, 16132 Genoa, Italy; 5 Infectious Diseases Unit, Ospedale Policlinico San Martino, IRCCS, 16132 Genoa, Italy; matteo.bassetti@hsanmartino.it (M.B.); daniele.roberto.giacobbe@gmail.com (D.R.G.); anton.vena@gmail.com (A.V.); 6 Department of Health Sciences (DISSAL), University of Genoa, 16132 Genoa, Italy; 7 Laboratory of Biochemical and Molecular Pharmacology, Institute of Biomedical Sciences, Federal University of Rio de Janeiro, Rio de Janeiro 21941-901, Brazil; silva.claudiamartins.ufrj@gmail.com; 8 Laboratory of Pulmonary Investigation, Carlos Chagas Filho Institute of Biophysics, Federal University of Rio de Janeiro, Rio de Janeiro 21941-901, Brazil; prmrocco@biof.ufrj.br

**Keywords:** COVID-19, thrombosis, coagulopathy, platelets, bleeding, heparin, acute distress respiratory syndrome

## Abstract

In critically ill patients with acute respiratory distress syndrome (ARDS) coronavirus disease 2019 (COVID-19), a high incidence of thromboembolic and hemorrhagic events is reported. COVID-19 may lead to impairment of the coagulation cascade, with an imbalance in platelet function and the regulatory mechanisms of coagulation and fibrinolysis. Clinical manifestations vary from a rise in laboratory markers and subclinical microthrombi to thromboembolic events, bleeding, and disseminated intravascular coagulation. After an inflammatory trigger, the mechanism for activation of the coagulation cascade in COVID-19 is the tissue factor pathway, which causes endotoxin and tumor necrosis factor-mediated production of interleukins and platelet activation. The consequent massive infiltration of activated platelets may be responsible for inflammatory infiltrates in the endothelial space, as well as thrombocytopenia. The variety of clinical presentations of the coagulopathy confronts the clinician with the difficult questions of whether and how to provide optimal supportive care. In addition to coagulation tests, advanced laboratory tests such as protein C, protein S, antithrombin, tissue factor pathway inhibitors, D-dimers, activated factor Xa, and quantification of specific coagulation factors can be useful, as can thromboelastography or thromboelastometry. Treatment should be tailored, focusing on the estimated risk of bleeding and thrombosis. The aim of this review is to explore the pathophysiology and clinical evidence of coagulation disorders in severe ARDS-related COVID-19 patients.

## 1. Introduction

The novel coronavirus (severe acute respiratory syndrome coronavirus-2, SARS-CoV-2), which emerged in late 2019 in Wuhan, China, is currently causing significant concern in the medical community because of its rapid global spread [1]. Since its identification in December 2019, the number of cases has risen to pandemic levels, and knowledge of the clinical and epidemiological features of this infection, known as coronavirus disease 2019 (COVID-19), is changing on a daily basis. Initial symptoms of COVID-19 include fever, myalgia, fatigue, and dry cough. Most severe cases develop dyspnea and hypoxemia within 1 week of disease onset, progressing quickly thereafter to an atypical acute respiratory distress syndrome (ARDS) or multiorgan failure [2,3]. However, there is growing evidence for an outstanding impact of cardiovascular events in COVID-19, especially in the most critical cases, with a high incidence of thromboembolic or hemorrhagic events [4,5,6].

Helms et al. reported a significantly higher risk of thromboembolic complications in COVID-19 ARDS patients when compared with non-COVID-19 ARDS (18% vs. 6%, odds ratio (OR) = 3.4 (95% confidence interval (CI) = 1.7–7.3), *p* < 0.001) [5].

A recent study [4] including 416 hospitalized patients with COVID-19 found that 19.7% had cardioembolic complications, and that these patients were at a higher risk of death (hazard ratio (HR), 4.26 (95% CI, 1.92–9.49)). In this study, the incidence of coagulation disorders was 2.9% in the overall population, with a higher rate (7.3 vs. 1.8%, *p* = 0.02) in patients with cardiac injury compared with those who did not present cardiological complications. High levels of interleukin (IL)-6, C-reactive protein (CRP), and D-dimers have been observed in patients with COVID-19 ARDS, and the magnitude of the activation of the inflammatory cascade seems to be strongly correlated with the severity of coagulation disorders. However, the risk factors, pathophysiology, and management of these complications are still poorly understood.

The aim of this manuscript is to explore the pathophysiology, clinical manifestations, and treatment of coagulation disorders in patients with ARDS-related COVID-19.

## 2. Pathophysiology of Coagulative Derangements in COVID-19 Patients

**Figure 1:**
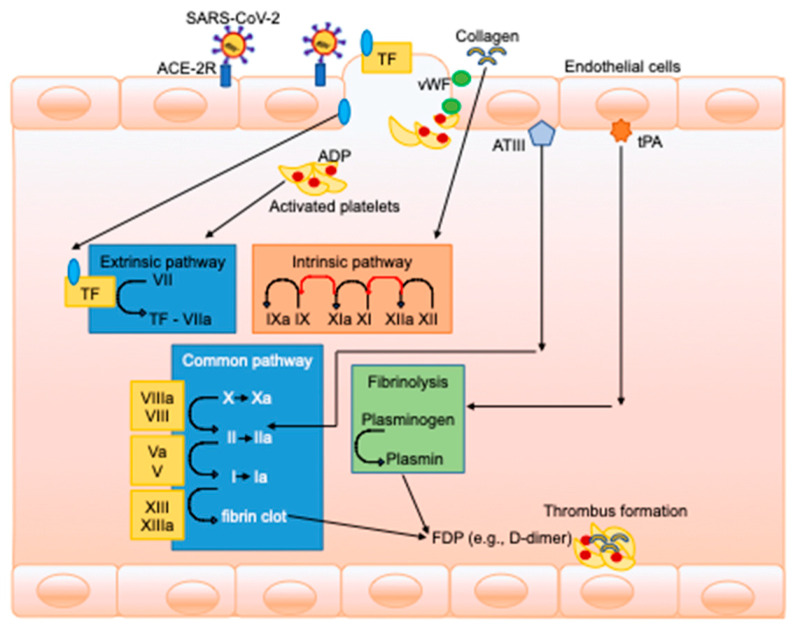
Coagulation cascade after severe acute respiratory syndrome coronavirus-2 (SARS-CoV-2) infection. Coagulation is initiated by viral binding to angiotensin-converting enzyme 2 receptors (ACE-2Rs) on endothelial cells, resulting in the exposure of tissue factor (TF) and collagen to blood and the release of von Willebrand factor (vWF). Platelets are exposed to TF, collagen, and vWF and activated, thus releasing mediators such as adenosine monophosphate (ADP) and vWF, leading to further platelet recruitment, followed by activation, aggregation, and plug formation. The TF–Factor VII (FVII) interaction activates the extrinsic pathway, while the exposed collagen starts the intrinsic pathway, helped by antithrombin-III (ATIII). Both pathways result in the common coagulation pathway, which ends with the formation of fibrin strands, thus leading to the formation of a stable platelet–fibrin clot. Meanwhile, tissue plasminogen activator (tPA) activates plasminogen to plasmin, which acts on the fibrin strands by degradation. ATIII, antithrombin-III; TF, tissue factor; tPA, tissue plasminogen activator; tPA, tissue plasminogen activator; vWF, von Willebrand factor; ADP, adenosine monophosphate; FDP, fibrin degradation products; ACE-2R, angiotensin-converting enzyme 2 receptors; SARS-CoV-2, severe acute respiratory syndrome coronavirus-2.

The hemostatic system consists of three phases (Figure 1) [7].

After an inflammatory trigger, the main mechanism for activation of the coagulation cascade is the tissue factor (TF) pathway. Through this pathway, endotoxin and tumor necrosis factor (TNF)-α trigger the production of interleukin (IL)-6 and IL-8 inhibitors, which is followed by an increase in thrombin and fibrin generation, thrombin–antithrombin complexes, and fibrinopeptide. Moreover, the fibrinolytic pathway is activated, potentially leading to an imbalance between activation of coagulation and regulation (activation/inhibition) of fibrinolysis [8]. The role of endothelial cell activation seems to be crucial in the development of shock and impairment of coagulation, and is a common feature of viral infection [9]. In fact, endothelial cells can be directly infected by several viruses, including adenoviruses, influenza, and herpes simplex virus [10].

SARS-CoV-2 may also downregulate angiotensin-converting enzyme 2 (ACE-2) expression, thus regulating overproduction of angiotensin II and concomitant enhancement of IL-6. IL-6 in a positive inflammatory feedback loop inactivates ACE-2, enhancing angiotensin II retention and leading to endothelial activation and inflammation [11].

A recent study on autopsies including seven lungs from patients who died from SARS-CoV-2 infection found three distinctive angiocentric features of ARDS-COVID-19: severe endothelial injury with disrupted endothelial cell membranes, widespread vascular thrombosis with microangiopathy, and occlusion of alveolar capillaries with significant new vessel growth through a mechanism of intussusceptive angiogenesis [12].

The net clinical manifestation may well be a procoagulant state, mainly by induction of TF expression on the endothelial surface in a process probably mediated by cytokines such as IL-1, TNF-α, and IL-6. During sepsis, the protein C/S system and thrombomodulin are also downregulated, resulting in a further decrease in protein C activity, thereby enhancing the procoagulant state. The change in endothelial cells from a resting to a procoagulant state may be associated with expression of endothelial surface adhesion molecules such as E-selectin and the von Willebrand factor, resulting in local inflammatory response, endothelial damage, and plasma leakage [8].

## 3. Understanding the Role of Platelets and Inflammation in Pneumonia Pathogenesis

The role of platelets is not only limited to the formation of the initial clot in the injured vessel endothelium to initiate hemostasis; platelets are also an integral part of the innate immune system, and act as proinflammatory cells [13,14,15]. Platelets can be activated by a number of mediators and cytokines that are stored within their alpha granules and whose release triggers further recruitment, activation, and aggregation of additional platelets in a downstream cascade [16,17,18,19]; once activated, they express a number of receptors for adhesion, clotting, and neutrophil activation. The consequent platelet–neutrophil aggregation process is responsible for local and systemic inflammation, especially in the lung, and can also generate reactive oxygen species (ROS), modulating the phagocytic capacity of neutrophils and formation of neutrophil extracellular traps [20,21]. The massive infiltration of activated platelets in the lungs that follows an insult or infection can be associated with thrombocytopenia, which is often seen in the course of many viral infections, albeit it is only occasionally serious enough to lead to hemostatic impairment and bleeding [22]. Although the mechanism of thrombocytopenia is mainly immune-mediated, decreased thrombopoiesis, increased platelet consumption, or a combination of both may also act as concomitant factors for its occurrence [22].

## 4. COVID-19 and Hyper/Hypocoagulable States

COVID-19 infection can activate the coagulation cascade through various mechanisms, leading to severe hypercoagulability, as SARS-CoV-2 infection is, in effect, a systemic disease not limited to the lungs. Initial clinical manifestations include fever, sore throat, fatigue, diarrhea, and other nonspecific symptoms [23,24]. During incubation (1–14 days, 3–7 days being more common), peripheral blood leukocytes and lymphocytes are not significantly reduced. Thereafter, the virus spreads, especially in tissues expressing ACE-2 (the SARS-CoV-2 receptor), i.e., the lungs, gastrointestinal tract, and heart. At this stage, pulmonary infiltration by blood cells increases, pulmonary lesions worsen, and chest computed tomography (CT) scans show peculiar imaging changes. Peripheral blood lymphocyte count (both T and B) significantly decreases, whereas inflammatory factors in the peripheral blood are raised [25]. In a previous study, Berri et al. [26] reported that plasminogen contributes to inflammation caused by influenza through fibrinolysis, and that 6-aminocaproic acid can protect against influenza. Presumably, fibrinolysis may also be induced following severe SARS-CoV-2 infection. High plasma levels of proinflammatory cytokines (IL-2, IL-7, granulocyte colony-stimulating factor, interferon-γ-induced protein-10, monocyte chemoattractant protein-1, macrophage inflammatory protein-1, and TNF-α) have been observed in patients with COVID-19 admitted to intensive care units, suggesting that a cytokine storm may be developing in individuals with severe disease [27]. Cytokine storm has been described as the primary cause of several disorders of immune dysregulation characterized by constitutional symptoms, systemic inflammation, and multiorgan dysfunction that can lead to multiorgan failure and coagulopathy if inadequately treated, with different severity and duration [27].

In the specific case of COVID-19, different factors can lead to thrombosis and to a hypercoagulable state. Among these, the need for quarantine or isolation reduces physical exercise and increases immobility and the risk for deep venous thrombosis. In agreement with this, high levels of IL-6, clotting activation, elevated D-dimer, and a high incidence of thromboembolic events have been reported [28]. Antiviral therapy itself—particularly ritonavir, which has been trialed for COVID-19 treatment in some centers—can lead to dysregulation of platelet function and prostaglandin-E2 production, and increases platelet aggregation [29]. Finally, the role of mechanical ventilation and positive intrathoracic pressure should be considered. In fact, mechanical ventilation itself can cause local and systemic inflammatory activation and a hypercoagulable state [30]. Positive end-expiratory pressure (PEEP) can also contribute to pulmonary vascular vasoconstriction, high pulmonary vascular resistance and pressures, and right-heart overload.

**Figure 2:**
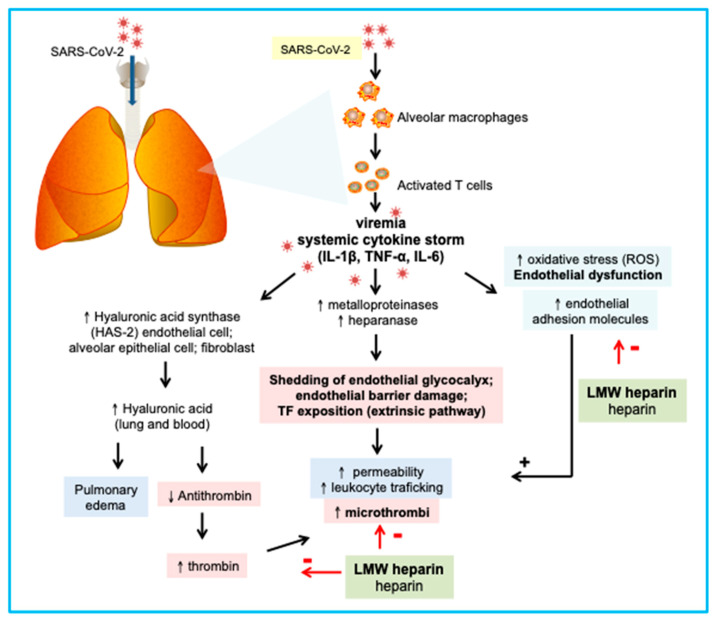
Potential beneficial roles of low molecular weight heparin (LMWH) on endothelial glycocalyx dysfunction during coronavirus disease 2019 (COVID-19). Figure Legend: − = inhibition; + = activation; ↑ = increase; ↓ = decrease.

Typical clotting derangements observed in COVID-19 include increased D-dimer (from mild to significant), prolonged prothrombin time (PT), and gradual decreases in fibrinogen and platelet counts [31,32]. Recent evidence has also shown that patients with COVID-19 who had poor outcomes exhibited ischemic clinical manifestations, such as mottling of the fingers and toes and organ dysfunction, thus suggesting a systemic hypercoagulable phase culminating in disseminated intravascular coagulation (DIC) [32]. DIC is an acquired disorder caused by hemostatic system stimulation, resulting in activation of platelets, activation of fibrinogen, and conversion of fibrinogen to fibrin, which triggers generalized microvascular thrombosis and life-threatening hemorrhage due to consumption of coagulation factors and activation of the fibrinolytic system. Hemorrhage may occur as a single clinical phenomenon or may be part of a more complex derangement of the coagulation cascade due to DIC or septic vasculitis or might even be iatrogenic as a result of anticoagulant treatment. In some cases of multi organ failure (MOF), bleeding and (microvascular) thrombosis may coexist. For the aforementioned reasons, early (perhaps universal) administration of low molecular weight heparin (LMWH) can be useful, whereas intravenous immunoglobulin (IVIG) may inhibit the development of cytokine storm or decrease its clinical impact [31,32]. Figure 2 represents a summary of the effect of heparin on endothelial function. However, attention should also be paid to the occurrence of hemorrhagic complications that often present as muscular and retroperitoneal bleeding and/or hemorrhagic shock.

During SARS-CoV-2 infection, alveolar macrophages and T cells (among other immune system cells) are recruited and activated. However, a hallmark of critically ill patients is an uncontrolled immune response leading to a cytokine storm characterized by increased serum levels of IL-1β, TNF-α, and IL-6. These cytokines reduce the expression of the constitutive endothelial nitric oxide synthase (eNOS) and increase the oxidative stress and the expression of adhesion molecules, thus favoring a dysfunctional endothelial phenotype. The glycocalyx is fundamental to epithelial and endothelial barrier function under homeostatic conditions and confers an anticoagulant and antiadhesive surface. Hyaluronan (or hyaluronic acid; HA) is a key component of glycocalyx. HA affects water homeostasis and excessive HA forms cable-like structures disrupting tissue architecture. IL-1β, TNF-α, and IL-6 are strong inducers of plasma-membrane-located HA synthase (HAS)-2 in endothelial cells, alveolar epithelial cells, and fibroblasts, and therefore HA may also participate in the pathophysiology of COVID-19. Moreover, HA inhibits antithrombin in vitro. If translated to the clinic, circulating HA may contribute to faster thrombin activity, explaining at least partially the coagulopathy found in critically ill patients, and the improvement mediated by low molecular weight heparin treatment. Moreover, IL-1β and TNF-α also target glycocalyx by activation of metalloproteinases and heparinize, consequently reducing the content of proteoglycans e.g., syndecan-1 and heparan sulfate, respectively. Glycocalyx shedding is linked to endothelial barrier rupture, which favors the increase of vascular permeability, leukocyte trafficking, and microthrombi. LMWH inhibits thrombin activity and protects endothelial cells from oxidative stress; therefore, it may counteract the consequences of cytokine storm during COVID-19. Furthermore, the use of steroids, which has been shown to improve outcome, can help in the minimization of the systemic inflammatory activation consequent to cytokine storm [33]. The role of other drugs with potential anti-inflammatory effects needs to be further investigated.

## 5. Clinical Evidence, Manifestations, and Treatment of Coagulative Disorders in COVID-19 Patients

Systemic infections may be complicated by activation of the coagulation cascade, with consequent subclinical and clinical manifestations ranging from a rise in laboratory markers to thromboembolic events and fulminant DIC [34]. Bleeding, thrombosis, or both may be the presenting clinical features [22]. In the 2002–2004 SARS epidemic, hematologic complications occurred in up to 63% of infected patients; activated partial thromboplastin time (aPTT) prolongation often developed within 2 weeks of infection, although some patients showed normal prothrombin time and no D-dimer elevation. Only 2.5% of SARS patients had DIC, but those affected were more likely to die [35].

In a study of 183 patients (41% with comorbidities, 11.5% mortality), coagulation parameters at hospital admission were comparable between survivors and non-survivors, except for PT, D-dimer, and fibrin degradation product (FDP) levels. Late in the hospital course, fibrinogen and antithrombin (AT) levels were also lower in non-survivors. The median time from admission to DIC onset was 4 days for those patients who died, while only 0.6% of those who were discharged from the hospital met the International Society on Thrombosis and Hemostasis (ISTH) diagnostic criteria for DIC [36].

A retrospective study of SARS-CoV-2-infected patients in Wuhan, China reported that patients with elevated markers of inflammation (e.g., high-sensitivity CRP and serum ferritin), coagulation (e.g., prothrombin and D-dimer), and end-organ damage (e.g., urea, lactate dehydrogenase, and aspartate transaminase (AST)) were at increased risk of developing severe ARDS and death. However, several other clinical factors—including comorbidities and cluster differentiation (CD3 and CD4 cell counts, AST, prealbumin, creatinine, ferritin, and prothrombin)—were in fact not associated with higher risk of death [37]. D-dimer values were higher in non-survivors as compared with survivors, suggesting DIC as the main mechanism of death in at least some patients [37]. These findings were confirmed by another small study of 109 COVID-19 patients. Patients who died showed higher rates of complications, including ARDS, acute cardiac injury, acute kidney injury, and DIC [38]. However, no cases of DIC were diagnosed in other cohorts of patients admitted to an Intensive Care Unit for ARDS due to COVID-19 [5,39], suggesting that hemostasis activation is not the same in all COVID patients, who can present with different phenotypes.

A recent retrospective study by Xu et al. [40] found that 16.67% of patients with COVID-19 were at high risk of venous thromboembolism (VTE) and 6.52% were at high risk of bleeding from VTE prophylaxis, with an incidence of deep venous thrombosis (DVT) among critically ill patients of 20%, which was even higher ( 69%) in a study by Litijos et al. [41].

Bompard et al. [42] in a retrospective study evaluating CT pulmonary angiographies found a total of 32 cases of pulmonary embolism, resulting in a 24% (95% CI; 17–32%) overall cumulative incidence; of those, 50% were in the intensive care unit. Similarly, in a cohort of 184 intensive care unit (ICU) patients, the cumulative incidence of symptomatic acute pulmonary embolism, deep venous thrombosis, acute ischemic stroke, myocardial infarction, and systemic arterial embolism was 49%.

Altogether, the evidence to date suggests that a variety of clinical features of coagulation derangement are present after viral infections and, in particular, in COVID-19, ranging from thromboembolic and hemorrhagic complications to DIC and vasculitis. The clinical course may be dominated by bleeding, thrombosis, or both, with a high rate of DVT or pulmonary embolism, which can result in life-threatening hemodynamic instability. Vasculitis triggered by infection may be present, resulting from infarction secondary to thrombotic occlusion of small blood vessels in the lung. Vasculitis may lead to ischemic injury (due to local occlusion) or bleeding (due to local tissue damage) [43,44]. The variety and complexity of clinical presentations of the coagulopathy syndromes confront the clinician with the difficult questions of whether, when, and how to provide optimal supportive care to restore the balance of coagulation. Furthermore, microthrombi in the pulmonary circulation can increase the areas that are aerated but not perfused, thus increasing shunt and causing hypoxemia. We believe that the clinician should be guided by whichever presenting symptom is most pronounced.

## 6. Coagulation Markers and Treatment Proposals

One of the key issues in the management of COVID-19 coagulopathy is the prompt recognition of hemostasis and coagulation disorders. In-ICU and post-ICU management should be individualized according to laboratory test results and clinical status. Current guidelines from different countries are showing similar shrewdness. Coagulation markers at hospital and ICU admission should include: D-dimers, PT and/or international normalized ratio (INR), aPTT, platelet count, and fibrinogen to monitor daily or twice daily relying on the preliminary results. Twice-daily monitoring should be considered in case of altered coagulation parameters at first glance: platelet count <100 × 10^9^/L, fibrinogen <2 g/L, and raised D-dimer (although a specific cutoff for D-dimer cannot be defined, a three to four-fold elevation is considered a markedly raised value) [44].

In non-bleeding patients, platelet count should be kept above 25 × 109/L; in bleeding patients, platelets above 50 × 109/L, fibrinogen above 1.5 g/L, and PT ratio <1.5 [44] should be maintained, as abnormal coagulation parameters are associated with poor outcome [45]. However, these also have several limitations and only partially reflect the coagulation balance [46].

Other tests include protein C, protein S, AT, tissue factor pathway inhibitor (TFPI), and coagulation factors (in particular factor Xa to monitor LMWH treatment). However, these are not often readily available for clinical use [47]. In this context, thromboelastography (TEG) and rotational thromboelastometry (ROTEM) are point-of-care tests that evaluate whole-clot formation and dissolution, enabling the assessment of different phases of the clotting process, and which could add valuable information on coagulation derangement to guide treatment strategies [48,49,50,51]. In a recent study assessing the role of rotation thromboelastometry, prothrombin time was slightly reduced, and it increased significantly after 10 days, whereas activated partial thromboplastin time and fibrinogen values were higher at admission. In general, thromboelastometry profiles presented a picture of hypercoagulability characterized by an acceleration of the propagation phase of blood clot formation and higher clot strength [49].

**Figure 3:**
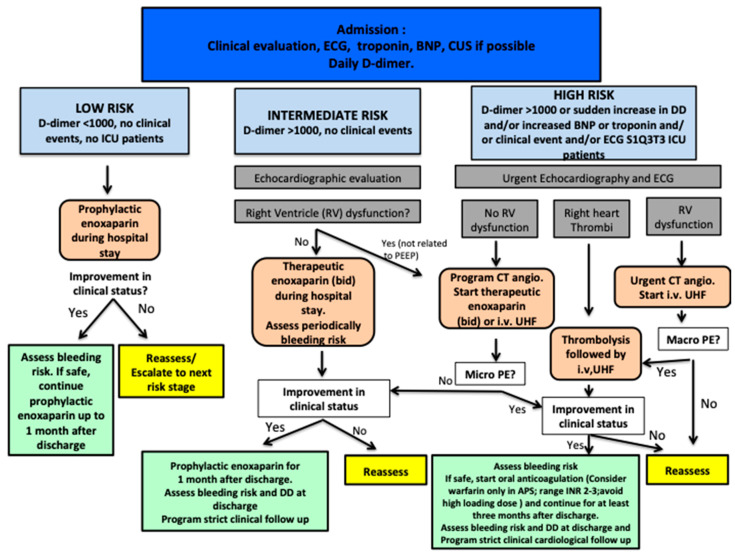
Algorithm of coagulation management in patients with COVID-19. We propose a strict monitoring of the risks/benefits of heparin administration in COVID-19 patients and the importance of coagulation control (risk of hemorrhage) not only in the hospital settings, but also after discharge. Heparin dose and administration during intensive care unit (ICU) stay should be evaluated on the basis of a laboratory test (in particular d-dimer, factor Xa, and thromboelastogram indices), and the occurrence of clinical events (local thrombosis, pulmonary embolism, bleeding, hemorrhagic shock). Prophylactic heparin can be continued after discharge under a strict follow up; in the most severe cases, oral anticoagulation should be taken in consideration after ICU stay. Abbreviations: PE, pulmonary embolism; UHF, unfractioned heparin, DD, D-dimer.

Although useful, TEG and ROTEM are not always easily available in ICUs, require special training of non-hematological staff members, and may be expensive [52]. Serial electrocardiogram, echocardiography, and lung ultrasound can also be useful for risk stratification. In Figure 3, we propose a treatment algorithm for coagulation management in COVID-19 patients, with three distinct clinical scenarios according to the risk of thrombosis and bleeding.

Recently, Ranucci et al. [53] described the typical procoagulant pattern of patients with COVID-19 acute respiratory distress syndrome as increased fibrinogen, an increased platelet count, and increased clot strength at viscoelastic tests that return to values close to normal after 14 days of aggressive anti-thrombotic therapy.

It is important to highlight that the risk/benefits of anticoagulation should be reassessed at each step of treatment, especially after discharge, considering the risk of hemorrhage and patients’ clinical status and clinical events (such as pulmonary embolism, venous thrombosis, local bleeding, and hemorrhagic shock) [40,44].

Considering the abovementioned pathophysiological features, blockade of platelet overactivation and aggregation can reduce the severity of lung impairment. In a mouse model, administration of the protease-activated-receptor-1 (PAR-1) antagonist SCH9797 (an inhibitor of thrombin-induced platelet activation) reduced inflammation [54]. Antiplatelet agents (such as clopidogrel, ticlopidine, and acetylsalicylic acid) may be considered according to the patient’s clinical condition. To reduce the number of microthrombi and the risk of pulmonary embolism, LMWH or unfractionated heparin (UFH) should be uniformly administered. However, important adverse effects have been reported in the literature after anticoagulant treatment, including major intramuscular bleeding (particularly of the iliopsoas) [55]. The recently published guidelines on the Diagnosis, Prevention, and Treatment of Venous Thromboembolism in Hospitalized Patients with COVID-19 recommend that practitioners use standard of care objective testing to diagnose VTE based on the clinical index of suspicion, whereas routine screening for VTE using bedside Doppler ultrasonography of the lower extremities or based on elevated D-dimer levels is not recommended [56].

Although the scientific community is waiting for more robust evidence in terms of treatment, guidelines suggest routine thromboprophylaxis with standard-dose UFH or LMWH after careful assessment of bleed risk, with LMWH as the preferred agent. Anticoagulant treatment has shown to be associated with decreased mortality in severe coronavirus disease patients with coagulopathy [45,57]. A position paper from the Italian Society on Thrombosis and Hemostasis (SISET) strongly reiterated that COVID-19 patients should be covered by LMWH, UFH, or fondaparinux at doses indicated for the prophylaxis of VTE for the entire duration of the hospital stay, and for 7–14 days more after hospital discharge [58]. Another Consensus Statement confirmed that in patients with active bleeding, temporarily contraindicating pharmacological prophylaxis, intermittent pneumatic compression should be adopted [57]. Moreover, in patients who show renal impairment (creatinine clearance <30 mL/min), UFH is preferred over LMWH [57]. The use of therapeutic doses of LMWH or UFH is currently not supported by evidence and cannot be recommended as a standard of care [57,58]. Finally, in cases of thrombocytopenia with suspicion of heparin-induced thrombocytopenia, danaparoid, argatroban, or bivalirudin may be preferred over rivaroxaban or fondaparinux [57].

While prophylaxis- and treatment-related criteria are fairly similar in all available guidelines, criteria for withholding anticoagulation do not fit all. Withholding of prophylactic anticoagulation with LMWH is proposed for platelet count <25 × 10^9^/L, platelet count <30–50 × 10^9^/L, or fibrinogen <1 g/L, while therapeutic anticoagulation should be held only if platelet count <25 × 10^9^/L or fibrinogen <0.5 g/L for the International Society on Thrombosis and Haemostasis – International Guidelines (ISTH-IG) recommendations; while the Scientific and Standardization Committee (SSC)-ISTH, Center for Diseases Control (CDC), and Anticoagulation Forum (ACF) guidelines do not suggest specific thresholds to withhold [59].

**Figure 4:**
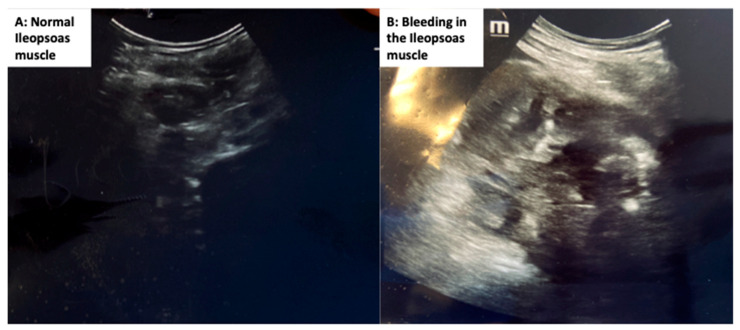
Ultrasound showing iliopsoas muscle bleeding in a patient from our institution.

In particular, resuming assisted and spontaneous breathing with potential asynchronies and coughing in patients under anticoagulant therapy may facilitate bleeding, in particular from sartorius and iliopsoas muscles (Figure 4). These two muscles are very soft and prone to hemorrhage, and therefore attention should be paid during physiotherapy (Figure 4). However, Paranjpe et al. found that among 2773 patients, 1.9% not receiving anticoagulant had bleeding events, compared with 3% who received anticoagulants (*p* = 0.2).

## 7. Conclusions

Coagulation abnormalities may have a major impact on the outcome of COVID-19 patients. A variety of clinical manifestations have been described in these patients, ranging from micro- to macrothrombi, with pulmonary embolism and hemodynamic instability requiring thrombolysis. Prophylactic anticoagulation and prompt diagnosis of complications are essential. However, treatment should be individualized on the basis of the patient’s risk of bleeding and thrombosis. Further data are needed in this setting and, more importantly, randomized controlled trials to evaluate the optimal treatment and management of coagulation—that represents one of the major issues to improve survival in COVID-19 patients.

## Data Availability

Not applicable.

## Abbreviations

ACE2	angiotensin converting enzyme 2
ARDS	Acute distress respiratory syndrome
aPTT	activated partial thromboplastin time
ARDS	acute respiratory distress syndrome
AST	aspartate transaminase
AT	antithrombin
CD	cluster differentiation
CI	confidence interval
CMV	cytomegalovirus
COVID-19	coronavirus disease 2019
CRP	C-reactive protein
CT	computed tomography
CVT	deep venous thrombosis
DIC	disseminated intravascular coagulation
FDPs	fibrin degradation products
HIV	human immunodeficiency virus
HR	hazard ratio
ICU	intensive care unit
IL	interleukin
INR	international normalized ratio
IVIG	intravenous immunoglobulin
LMWH	low molecular weight heparin
MOF	multiorgan failure
OR	odds ratio
PEEP	positive end-expiratory pressure
PT	prothrombin time
R	reaction time
ROS	reactive oxygen species
ROTEM	rotational thromboelastometry
rPA	tissue plasminogen activator
SARS-CoV-2	severe coronavirus disease-2
TEG	thromboelastography
TF	tissue factor
TFPI	tissue factor pathway inhibitor
TNF-α	tumor necrosis factor-α
UFH	unfractionated heparin
VTE	venous thromboembolism
vWF	von Willebrand factor
